# Surface Wipe Sampling of Hazardous Medicinal Products: A European Interlaboratory Comparison Study

**DOI:** 10.1002/dta.3902

**Published:** 2025-05-05

**Authors:** Roland B. van den Berg, Ewelina Korczowska, Mónica S. F. Santos, Maria Francisca Portilha‐Cunha, Ana R. L. Ribeiro, Lucie Bláhová, Luděk Bláha, Claudia Vom Eyser, Jochen Tuerk, Richard C. J. M. van Rossen, Erik B. Wilms, Mirjam Crul

**Affiliations:** ^1^ Department of Pharmacy and Clinical Pharmacology, Amsterdam UMC Vrije Universiteit Amsterdam Amsterdam the Netherlands; ^2^ Department of Hospital Pharmacy Haaglanden Medisch Centrum The Hague the Netherlands; ^3^ Department of Hospital Pharmacy University Clinical Hospital Poznan Poland; ^4^ EPIUnit ITR, Institute of Public Health of the University Porto University of Porto Porto Portugal; ^5^ LEPABE—Laboratory for Process Engineering, Environment, Biotechnology and Energy, Faculty of Engineering University of Porto Porto Portugal; ^6^ ALiCE—Associate Laboratory in Chemical Engineering, Faculty of Engineering University of Porto Porto Portugal; ^7^ Laboratory of Separation and Reaction Engineering—Laboratory of Catalysis and Materials (LSRE‐LCM), Faculty of Engineering University of Porto Porto Portugal; ^8^ RECETOX, Faculty of Science Masaryk University Brno Czech Republic; ^9^ Institut für Umwelt & Energie, Technik & Analytik e. V. (IUTA) Duisburg Germany; ^10^ Instrumental Analytical Chemistry University of Duisburg‐Essen Essen Germany; ^11^ Centre for Water and Environmental Research (ZWU) University of Duisburg‐Essen Essen Germany; ^12^ Apotheek Haagse Ziekenhuizen The Hague the Netherlands; ^13^ Department of Hospital Pharmacy Haga Teaching Hospital The Hague the Netherlands

**Keywords:** analytical method, antineoplastic drugs, quality control, surface contamination, workplace monitoring

## Abstract

Workplace monitoring of hazardous medicinal products (HMPs) using surface wipe sampling is becoming common practice in many European hospitals and pharmacies. However, no independent quality control is available to validate wiping procedures and analytical methods. This study aimed to conduct a Europe‐wide interlaboratory comparison (ILC) program to independently and blindly assess laboratory performance and variability in HMP detection. Four European laboratories participated in the study. Six HMPs—cyclophosphamide, etoposide, gemcitabine, ifosfamide, methotrexate, and paclitaxel—were prepared at four concentrations (5000, 2000, 200, and 20 ng/mL) and applied to a 400‐cm^2^ stainless‐steel surface, then wiped by the coordinating body according to each laboratory's protocol. Wipe samples were distributed to individual laboratories, where blind analyses were conducted. Target criteria for accuracy and recovery were set at 70%–130% and 50%–130%, respectively. Of the 80 samples, 69 (86%) met accuracy targets, and 70 (88%) met recovery targets. Accuracy was often overestimated for the lowest concentrations of cyclophosphamide, etoposide, methotrexate, and paclitaxel by Laboratory A. Laboratory D showed low accuracy for paclitaxel at three lower concentrations. Among the 10 samples that did not meet recovery targets, all were below 50% and involved etoposide and paclitaxel. This ILC program demonstrates a viable method for evaluating laboratory performance in HMP detection, offering an external validation mechanism for surface wipe sampling methods. A future goal is to establish a global ILC program with a designated coordinating body for managing it effectively.

## Graphical abstract

This study aimed to conduct an interlaboratory comparison (ILC) program to evaluate the performance of European laboratories in detecting hazardous medicinal products (HMPs) from surface wipe samples, using their own sampling and analytical methods. Results showed that accuracy was achieved in 69 of 80 concentrations (86%) and recovery in 70 of 80 concentrations (88%). The ILC program demonstrated a reliable approach for assessing laboratory performance in HMP detection.

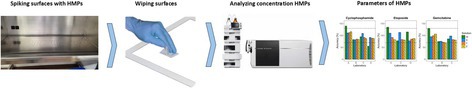

## 1 Introduction

Workplace monitoring of hazardous medicinal products (HMPs) with surface wipe sampling is becoming a standard procedure in many European hospitals and pharmacies, in accordance with European Guidance [1] and the European Guidelines QuapoS [2]. Surface wipe sampling offers the possibility for monitoring the level of contamination, potential occupational exposure and cleaning efficacy in compounding facilities and wards of hospitals. Direct contact with HMPs without personal protective equipment is recognized as a major source of unintentional exposure [3]. Occupational exposure can differ depending on the tasks performed by healthcare workers, as well as their working conditions and education levels [4, 5, 6]. Moreover, it is acknowledged that the type of detergent or disinfectant used, as well as the cleaning procedure, can further influence occupational exposure [7]. Although official limits for surface contamination with HMPs are yet to be established within existing regulatory frameworks, surface contamination with HMPs in pharmacies seems to have decreased over time [8, 9]. Nevertheless, the regular determination of HMPs using surface wipe sampling remains a powerful methodology for estimating occupational exposure and associated risks [10], as well as guiding the implementation of risk control measures to ensure healthier and safer working conditions. When periodically repeating surface wipe sampling, it is possible to perform longitudinal measurements or benchmarks to determine safe reference values of surface contamination with alert and action levels [9, 11, 12] and ultimately contribute to the definition of official limits for surface contamination with HMPs.

To assess the performance of methods (sampling, sample preparation and extraction, and instrumental analysis) independently, accredited laboratories are expected to organize and participate in interlaboratory comparison (ILC) investigations according to “ISO/IEC 17025:2017: General requirements for the competence of testing and calibration laboratories” or “ISO 15189:2022: Medical laboratories ‐ Requirements for quality and competence” [13, 14]. Although ILCs exist for some types of analyses (e.g., assessment of drugs in biological matrices [15, 16]), reports on the analysis of HMPs extracted from surface wipe samples with validated methods [17, 18, 19] are still limited. Therefore, conducting an ILC of surface wipe sampling of HMPs remains challenging. Furthermore, no reference matrices for surface wipe sampling are available to enable the validation of the same method across different laboratories, primarily due to variations in surface wipe sampling protocols among laboratories, including differences in the material of the wipe, the solvent used during wiping, and the wiping strategy employed. To the best of our knowledge, no protocol was previously established for conducting an ILC investigation with surface wipe sampling of HMPs.

The aim of the study was to conduct an ILC program that, for the first time, evaluated the performance of different European laboratories—utilizing their own sampling protocols and analytical methods—and assess the variability associated with the results obtained under controlled blinded circumstances.

## 2 Materials and Methods

### 2.1 Outcome

The primary outcome of the study was the concentration of six HMPs in a mixed analytical standard—cyclophosphamide, etoposide, gemcitabine, ifosfamide, methotrexate, and paclitaxel—reported by the participating laboratories. The inclusion criteria for HMPs required that the compounds be amenable to collection and analysis via a single surface wipe sampling procedure. The selection of HMPs was guided by the validated analytical methods available in the participating laboratories, ensuring compatibility with single‐wipe sampling. Furthermore, the selected HMPs needed to exhibit solubility in a mixture of acetonitrile and water to facilitate the preparation of a unified stock solution containing all selected compounds.

### 2.2 ILC Program of Surface Wipe Sampling

To evaluate the performance of European laboratories in determining surface contamination by antineoplastic drugs, using their own surface wipe sampling protocols and analytical methods, and to assess the variability of the results, an ILC was conducted as a proof of concept among four laboratories with established methods for measuring HMPs from surface wipe samples. The four participating laboratories (A, B, C, and D), each having a validated method for analyzing at least three of the selected HMPs, sent their surface wipe sampling kits to the coordinating body. The validated methods for each laboratory are presented in Table S1. Three researchers, unaffiliated with any of the participating laboratories, served as the coordinating body and conducted surface wipe sampling at a laboratory in the Netherlands. At this central laboratory, the surface wipe sampling was carried out using the received kits and protocols from each participating laboratory on a stainless‐steel surface within a laminar airflow cabinet. The entire surface was cleaned three times using 0.05‐M sodium hydroxide and isopropyl alcohol alternately, both before spiking the areas. This decontamination protocol was consistently applied before each spiking procedure for each laboratory. Sodium hydroxide was employed as a cleaning agent due to its ability to decompose cytotoxic drugs, while isopropyl alcohol was utilized owing to its high effectiveness, exceeding 80%, in removing contamination from surfaces commonly exposed to cytotoxic drugs [20, 21].

**FIGURE 1:**
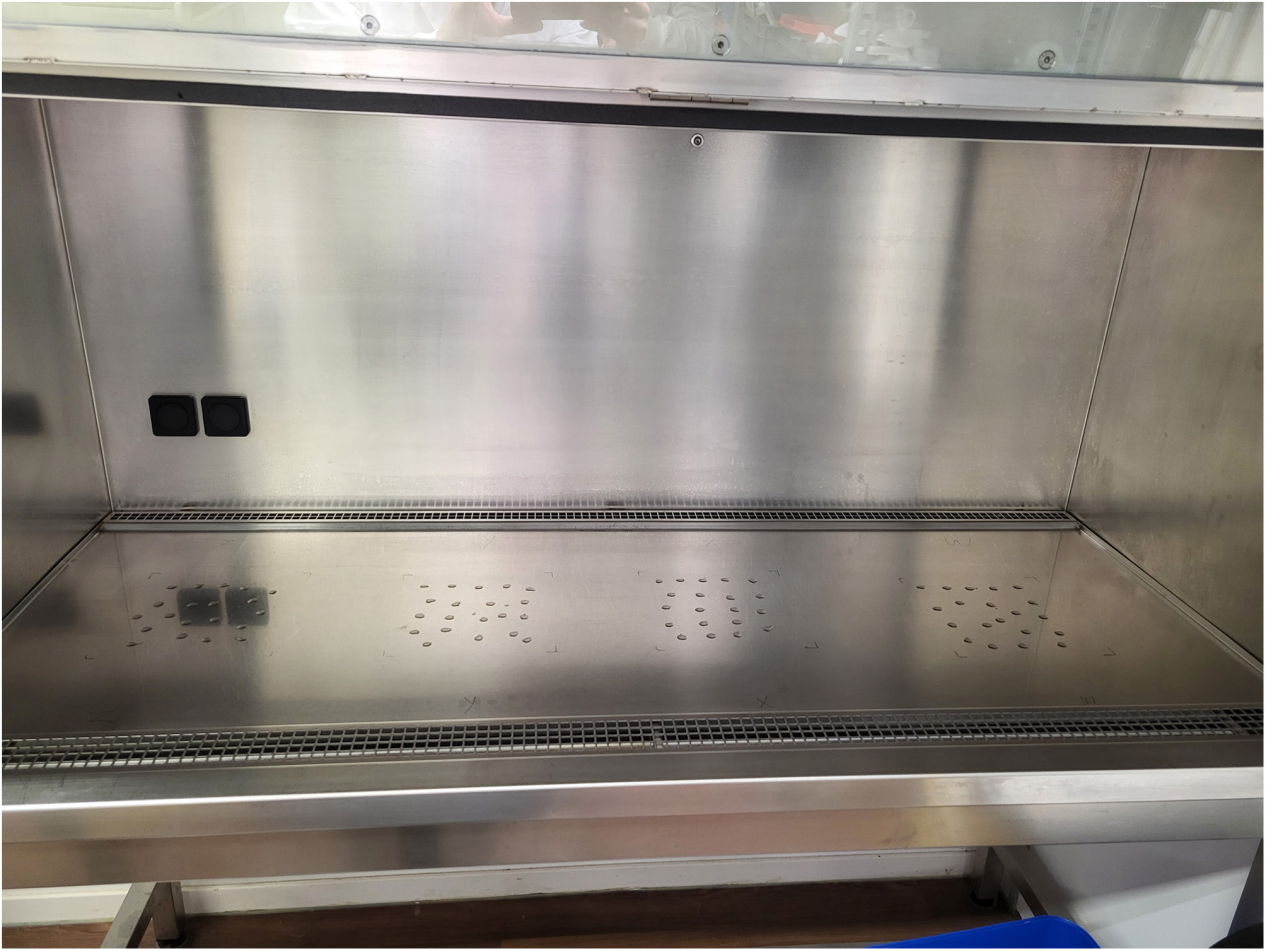
Illustration of the sampling strategy, wherein a stainless‐steel surface was divided into four areas, each measuring 20 by 20 cm (400 cm^2^), labeled as Areas W, X, Y, and Z. Each area was spiked with 1 mL of an acetonitrile–water mixture (1:1, v/v) or 1 mL of solution containing different concentrations of a mixture of HMPs (Solution W, X, Y, or Z). The 1‐mL volume was evenly distributed across each area in the form of 20 small drops.

Subsequently, the surface was divided into four areas, each measuring 20 by 20 cm (400 cm^2^), labeled as Areas W, X, Y, and Z. Each area was initially spiked with 1 mL of an acetonitrile–water (blank) mixture (1:1, v/v) by evenly distributing the 1 mL into 20 small drops as shown in Figure 1 by using calibrated electronic volumetric pipettes (Mettler‐Toledo International Inc., Columbus, Ohio, USA). After the drops had completely evaporated, the four areas were wiped. These served as blank samples to ensure baseline measurements of surface contamination (SWS‐BW, SWS‐BX, SWS‐BY, and SWS‐BZ). Next, the four areas were spiked with 1 mL of the corresponding Solutions W, X, Y, or Z, again evenly distributing the 1 mL into 20 small drops (as described in the following paragraph). After these drops evaporated, the areas were wiped again to obtain contaminated surface wipe samples (SWS‐W, SWS‐X, SWS‐Y, and SWS‐Z). All SWS was performed using the laboratory's surface wipe sampling protocols and materials. For each laboratory, the blank samples were conducted first, followed by spiked samples. All the wiping for both blank and spiked samples were conducted per participating laboratory on different days. The surface wipe samples, consisting of four blanks and four contaminated samples per laboratory, along with 1 mL each of Solutions W, X, Y, and Z, were transported on the same day and arrived within 24 h at the respective participating laboratory after conducting surface wipe sampling. The samples were stored at 2°C–8°C during transportation. Samples were analyzed within 1 month of preparation, with the exception of one laboratory, where technical issues with the LC–MS/MS system resulted in the analysis being conducted after 6 months. This study did not require ethical approval from a medical research ethics committee, as it does not involve human participants, their data, or biological material.

### 2.3 Chemicals, Stock Solution, and Diluted Solutions

Chemical reference substances (CRS) of cyclophosphamide, etoposide, gemcitabine, ifosfamide, methotrexate, and paclitaxel (European Directorate for the Quality of Medicines & HealthCare, Strasbourg, France), as presented in Table S2, and acetonitrile–water mixture (1:1, v/v), prepared using acetonitrile (Merck KGaA, Darmstadt, Germany) and water (ELGA LabWater, High Wycombe, United Kingdom), were utilized to prepare the stock solution. Each stock solution was prepared by accurately weighing 10 mg of each CRS using a calibrated milligram balance, type XPE105/M (Mettler‐Toledo International Inc., Columbus, Ohio, USA). In the case of cyclophosphamide, etoposide, gemcitabine, ifosfamide, and methotrexate, they were dissolved in 5 mL of acetonitrile–water (1:1, v/v), resulting in a concentration of 2 mg/mL. Paclitaxel was dissolved in 4 mL of acetonitrile–water (1:1, v/v) and 1 mL of dimethyl sulfoxide (Thermo Fisher Scientific, Waltham, Massachusetts, USA), resulting in a concentration of 2 mg/mL. The accuracy of the concentrations of the liquid samples was ensured through precise weighing of the CRS for HMPs and dissolution in calibrated volumetric flasks (VWR International, Radnor, Pennsylvania, Verenigde Staten) using calibrated positive displacement pipettes (Gilson Inc., Middleton, Wisconsin, USA) and air displacement pipettes (Eppendorf SE, Hamburg, Germany) according to a predetermined protocol, executed by an experienced laboratory technician in an ISO 15189 accredited laboratory.

Next, 250 μL of each 2‐mg/mL solution was diluted to 5 mL with acetonitrile–water (1:1, v/v), resulting in a concentration of 100 μg/mL. Subsequently, 100 mL of a solution containing all the six HMPs at a final concentration of 5000 ng/mL (referred to as Solution Z) was prepared by diluting 5 mL of each diluted solution in acetonitrile–water (1:1, v/v). Solution Z, containing all six HMPs at a concentration of 5000 ng/mL, was then diluted to concentrations of 2000 (Solution Y), 200 (Solution X), and 20 ng/mL (Solution W) using as solvent the acetonitrile–water mixture (1:1, v/v), prepared using acetonitrile (Biosolve, Dieuze, France) and water (Veolia Water Technologies Netherlands, Ede, the Netherlands). Dilution from the stock solution to the four concentrations was performed in calibrated volumetric flasks (Hirschmann Laborgeräte GmbH & Co. KG, Eberstadt, Germany) using calibrated pipettes (BRAND GMBH + CO KG, Wertheim, Germany), executed by an experienced laboratory technician in an ISO 15189 accredited laboratory. The concentration range of 20–5000 ng/mL was selected to encompass values below, between, and above the established alert and action limits of 0.1 and 10 ng/cm^2^, respectively. Specifically, a concentration of 20 ng/mL corresponds to 0.05 ng/cm^2^, which is twice below the alert limit, while a concentration of 5000 ng/mL corresponds to 12.5 ng/cm^2^, exceeding the action limit by 25%.

### 2.4 Analysis of HMPs and Parameters

Each laboratory received the following samples: the four diluted solutions (Solutions W, X, Y, and Z), four contaminated surface wipe samples (SWS‐W, SWS‐X, SWS‐Y, and SWS‐Z) and four blank surface wipe samples (SWS‐BW, SWS‐BX, SWS‐BY, and SWS‐BZ). After receiving the samples, each participating laboratory conducted analyses using their own validated methods [7, 17, 19] and samples were processed and analyzed in accordance with laboratory's standard protocols. Laboratories were asked to handle and analyze the samples as if they were regular submitted samples. Two of the four laboratories had validated methods for analyzing all selected HMPs. Consequently, these laboratories were capable of determining six concentrations per sample, resulting in a total of 24 concentrations across all sample types, including liquid samples, surface wipe samples, and blank surface wipe samples. The remaining two laboratories had validated methods for analyzing only four of the six HMPs, allowing them to determine four concentrations per sample, leading to a total of 16 concentrations across all sample types. Throughout the study, all participating laboratories were blinded to the specific concentrations of the HMPs used. The laboratories were provided with a case report form to report the concentrations of the four solutions (in ng/mL) and the contamination levels of the surface wipe samples (in ng/400 cm^2^). The results from the four laboratories were compiled, and accuracy, recovery, and precision were calculated and evaluated for each laboratory as primary parameters. Precision was calculated and evaluated for each laboratory as secondary parameter. Accuracy was assessed based on the concentrations reported for the four solutions (W, X, Y, and Z), while recovery was calculated from the reported concentrations of the wipe samples. Accuracy was defined as the percentage of the predetermined concentration in ng/mL. Recovery was calculated as a percentage of the predefined concentration in the contaminated surface wiped (in ng/400 cm^2^). This approach of calculating the recovery reflects all the steps of the analytical method, combining the process of HMP extraction from the surface (i.e., absorption into the wipe), its subsequent extraction into the solvent, and the instrumental analysis. Precision was evaluated based on the relative standard deviation (RSD) derived from triplicate analyses of the same analytical standard. The RSD was calculated by dividing the standard deviation by the mean.

In Europe, while there are guidelines that provide general principles for the validation of analytical methods, such as ISO/IEC 17025 and the European Commission Decision 2002/657/EC, universally fixed values for parameters like recovery, accuracy, precision, and linearity are not explicitly established. Instead, laboratories are encouraged to define acceptance criteria based on the specific nature of the method, the analyte, and its intended application. Regarding the concentration parameters of HMPs extracted from surface wipe samples, no predefined acceptable performance criteria had been established previously. Therefore, the target performance criteria were defined following consultation of the participation laboratories to assess the rationale and feasibility of these criteria. This consultation also considered the years of experience of the participating laboratories and relevant literature on achieved results regarding recovery, accuracy, and precision [19]. This resulting in a consensus among the four participating laboratories and led to the following target performance criteria: accuracy within 70%–130%, recovery between 50% and 130%, and precision ≤ 20%. The recovery reflects a combined measure of both accuracy and precision. Consequently, an upper limit of 130% was predetermined as the acceptable threshold. Further, the recovery lower limit has been set on 50%, as this parameter reflects the capability of both the absorption of HMPs during the wiping procedure and the extraction from the wipe during the extraction process.

## 3 Results

Among the four laboratories that participated in this study, two laboratories (Laboratories A and C) had validated methods for analyzing all six HMPs. Laboratory B had a validated method for the detection of cyclophosphamide, gemcitabine, ifosfamide, and methotrexate, while Laboratory D had a validated method for cyclophosphamide, etoposide, ifosfamide, and paclitaxel. A total of 16 liquid samples, 16 blank samples, and 16 spiked samples were prepared, collected, and analyzed that resulted in 80 concentrations of HMPs derived from the liquid samples, 80 concentrations of HMPs derived from the blank samples, and 80 concentrations of HMPs derived from the spiked samples. All 80 concentrations derived from blank samples are presented in Table S3 and were reported as either non‐detectable or below the lower limit of quantification of the analytical method used.

### 3.1 Accuracy

**FIGURE 2:**
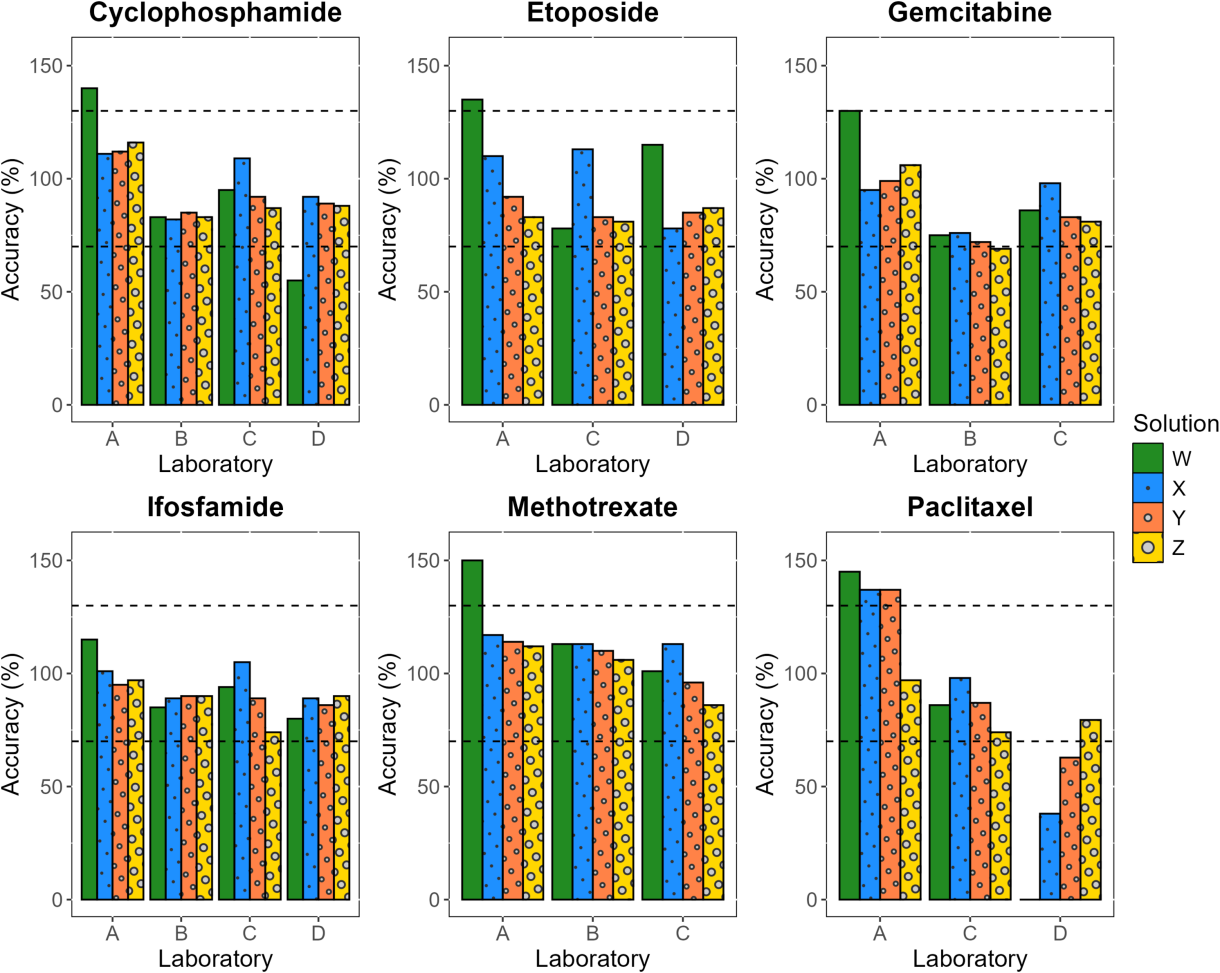
Accuracy per HMP (cyclophosphamide, etoposide, gemcitabine, ifosfamide, methotrexate, and paclitaxel) and per laboratory (A, B, C, and D) for the four concentrations evaluated: 20 (Solution W, in green), 200 (Solution X, in blue), 2000 (Solution Y, in orange), and 5000 ng/mL (Solution Z, in yellow). The dashed horizontal lines represent the limits of 70% and 130%.

Of the 80 concentrations derived from liquid samples, 69 (86%) met the target accuracy, as presented in Figure 2 and Table S4. Laboratory A exhibited excessively high concentrations, exceeding the accuracy limit of 130%, for six concentrations and specifically for cyclophosphamide, etoposide, methotrexate, and paclitaxel. For cyclophosphamide, etoposide, and methotrexate, only the lowest concentration (Solution W) exceeded the target, while for paclitaxel, the three lowest concentrations surpassed the target. The other deviations in accuracy fell below the target, including Solution W of cyclophosphamide (Laboratory D); Solution Z of gemcitabine (Laboratory B); and Solutions W, X, and Y of paclitaxel (Laboratory D).

### 3.2 Recovery

**FIGURE 3:**
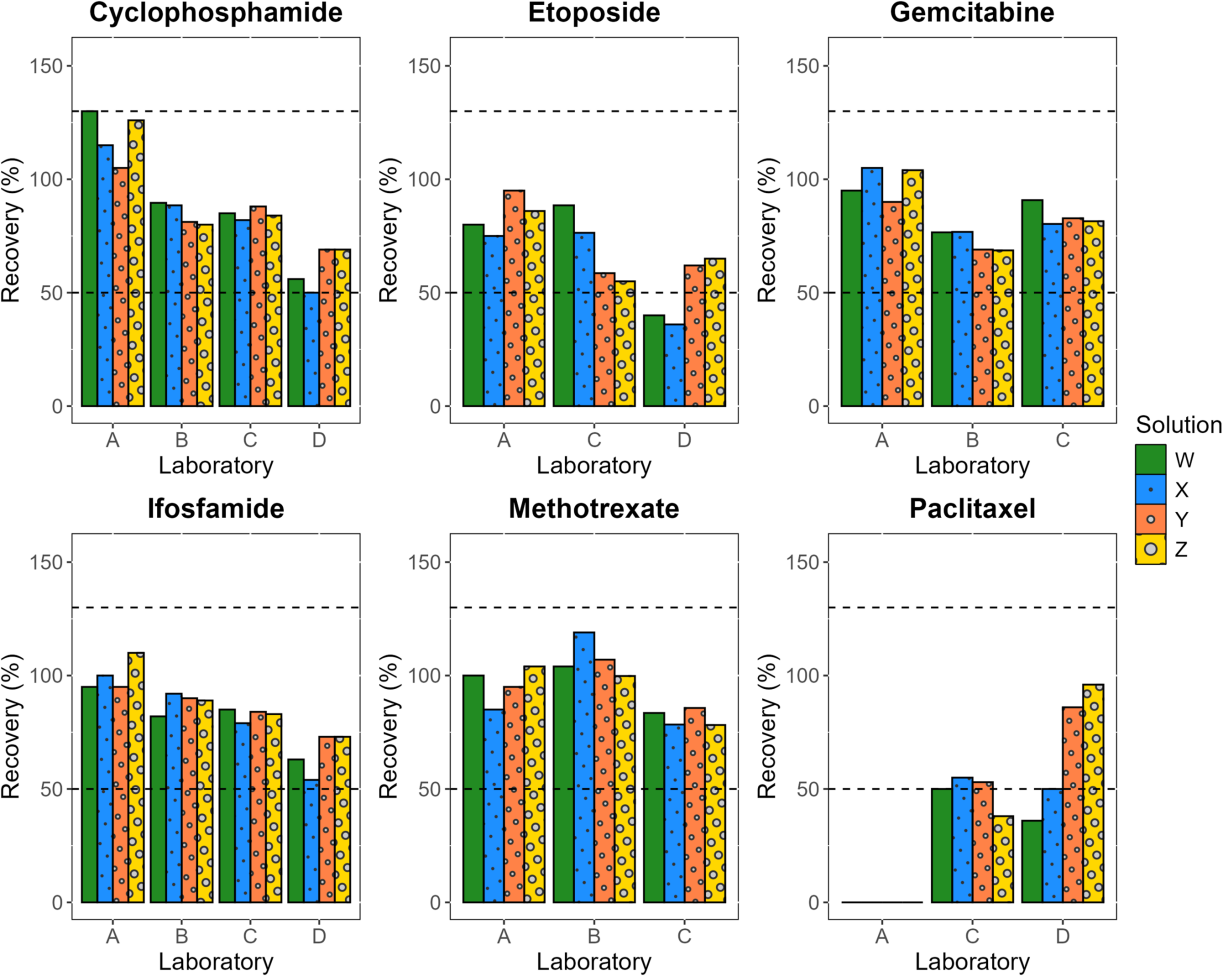
Recovery per HMP (cyclophosphamide, etoposide, gemcitabine, ifosfamide, methotrexate, and paclitaxel) and per laboratory (A, B, C, and D) for the four concentrations, extracted from the wipe, evaluated: 0.05 (SWS‐W, in green), 0.5 (SWS‐X, in blue), 5 (SWS‐Y, in orange), and 12.5 ng/cm^2^ (SWS‐Z, in yellow). The dashed horizontal lines represent the limits of 50% and 130%.

The recovery met the target in 70 out of 80 concentrations (88%), as illustrated in Figure 3 and Table S5. Of the 10 concentrations that did not meet the target recovery, all had a recovery below 50%. This was observed in etoposide (two concentrations) and paclitaxel (eight concentrations) from Laboratories A, C, and D (Laboratory B did not analyze paclitaxel). Additionally, the concentration of paclitaxel determined by Laboratory A was 0% for all solutions. Contrarily, one concentration (1%) had a recovery above 130%, which was observed with cyclophosphamide in Solution W, determined by Laboratory A.

### 3.3 Precision

Precision was calculated only for HMPs analyzed by Laboratories B, C, and D, as these laboratories performed triplicate analyses of the same sample. Laboratory A inadvertently conducted single analyses (singlicate). Therefore, the calculation of the RSD based on triplicate measurements of the same sample was not possible for the HMPs analyzed by Laboratory A. The precision target was achieved in 53 of the 55 concentrations derived from the liquid samples (96%) and 55 of the 56 concentrations derived from surface wipe samples (98%), as depicted in Figures S1 and S2 and presented in Table S6. The three concentrations that exceeded the 20% precision target were paclitaxel, as determined by Laboratory D (two concentrations derived from liquid samples and one concentration derived from surface wipe sample).

### 3.4 Overall Performance

The accuracy of Laboratory A was achieved in 18 out of 24 concentrations (75%), in 15 out of 16 concentrations (94%) for Laboratory B, in all 24 concentrations (100%) for Laboratory C, and in 12 out of 16 concentrations (75%) for Laboratory D. The recovery for Laboratory A was achieved in 20 out of 24 concentrations (83%), in 16 out of 16 concentrations (100%) for Laboratory B, in 22 out of 24 concentrations (92%) for Laboratory C, and in 12 out of 16 concentrations (75%) for Laboratory D. The precision of Laboratory B was demonstrated in all 32 concentrations derived from liquid and surface wipe samples (100%), in all 48 concentrations derived from liquid and surface wipe samples (100%) for Laboratory C, and in 28 out of 31 concentrations derived from liquid and surface wipe samples (90%) for Laboratory D. The performance per laboratory per HMP is displayed in Table 1, where also the overall performance per laboratory is given.

**TABLE 1:** Performance of the accuracy, recovery, and precision per HMP (cyclophosphamide, etoposide, gemcitabine, ifosfamide, methotrexate, and paclitaxel) and per laboratory (A, B, C, and D) including the overall performance per laboratory.

HMP	Parameter	Laboratory A	Laboratory B	Laboratory C	Laboratory D
Cyclophosphamide	Accuracy (%)	3/4 (75%)	4/4 (100%)	4/4 (100%)	3/4 (75%)
	Recovery (%)	4/4 (100%)	4/4 (100%)	4/4 (100%)	4/4 (100%)
	Precision (%)	n.a.	8/8 (100%)	8/8 (100%)	8/8 (100%)
Etoposide	Accuracy (%)	3/4 (75%)	n.a.	4/4 (100%)	3/4 (75%)
	Recovery (%)	4/4 (100%)	n.a.	4/4 (100%)	2/4 (50%)
	Precision (%)	n.a.	n.a.	8/8 (100%)	8/8 (100%)
Gemcitabine	Accuracy (%)	4/4 (100%)	3/4 (75%)	4/4 (100%)	n.a.
	Recovery (%)	4/4 (100%)	4/4 (100%)	4/4 (100%)	n.a.
	Precision (%)	n.a.	8/8 (100%)	8/8 (100%)	n.a.
Ifosfamide	Accuracy (%)	4/4 (100%)	4/4 (100%)	4/4 (100%)	4/4 (100%)
	Recovery (%)	4/4 (100%)	4/4 (100%)	4/4 (100%)	4/4 (100%)
	Precision (%)	n.a.	8/8 (100%)	8/8 (100%)	8/8 (100%)
Methotrexate	Accuracy (%)	3/4 (75%)	4/4 (100%)	4/4 (100%)	n.a.
	Recovery (%)	4/4 (100%)	4/4 (100%)	4/4 (100%)	n.a.
	Precision (%)	n.a.	8/8 (100%)	8/8 (100%)	n.a.
Paclitaxel	Accuracy (%)	1/4 (25%)	n.a.	4/4 (100%)	1/4 (25%)
	Recovery (%)	0/4 (0%)	n.a.	2/4 (50%)	2/4 (50%)
	Precision (%)	n.a.	n.a.	8/8 (100%)	4/7^a^ (57%)
Overall performance	Accuracy (%)	18/24 (75%)	15/16 (94%)	24/24 (100%)	12/16 (75%)
	Recovery (%)	20/24 (83%)	15/16 (100%)	22/24 (92%)	12/16 (75%)
	Precision (%)	n.a.	32/32 (100%)	48/48 (100%)	28/31 (90%)

Abbreviation: n.a., not applicable.

^a^ The concentrations obtained from the triplicate analyses of Solution W were below the limit of quantification, making it impossible to calculate the RSD. Therefore, the precision could only be determined using seven samples instead of eight.

## 4 Discussion

This study demonstrated that this ILC program is capable of independently evaluating the analytical methods for assessing HMPs on surfaces. The findings from these ILC assessments provide insights into whether the analysis of HMPs meets defined standards for accuracy and recovery. Although the overall performance, aggregated across the assessed HMPs, ranged between 75% and 100% across the three evaluated parameters, none of the participating laboratories met all performance criteria for all HMPs, as presented in Table 1. Nevertheless, the failure to meet all performance criteria for every HMP does not necessarily indicate that the data generated by these laboratories concerning HMP surface contamination are without value for healthcare institutions undertaking surface wipe sampling. As previously noted, the primary utility of these results lies in indicating whether healthcare organizations maintain adequate control over contamination or whether elevated contamination levels warrant targeted cleaning measures and intensified monitoring efforts. Moreover, the results obtained do not directly reflect occupational exposure, as there is no established correlation between surface contamination levels and the presence of HMPs in the urine of healthcare workers [22]. Furthermore, in terms of accuracy and recovery, Laboratory A met the performance criteria for two of the six HMPs, Laboratory B for three of four, Laboratory C for five of six, and Laboratory D for one of four (Table 1). For these specific analytical methods, the participating laboratories successfully met the corresponding parameter criteria.

In addition to assessing the performance of analytical methods for the determination of HMPs on surfaces, this ILC program also serves to identify specific areas for improvement in the methodology for each HMP of participating laboratories, thereby contributing to the enhancement of overall analytical quality. Moreover, the routine implementation of such an ILC program, combined with the transparent reporting of individual laboratory performance for each HMP enables healthcare organizations to more effectively identify laboratories that meet the required quality standards for analyzing surface contamination. Additionally, it also facilitates the identification of specific analytical methods for individual HMPs that fail to meet one or more performance criteria. This, in turn, ensures which laboratories are capable of detecting contamination levels for specific HMPs that align with the established alert and action limits.

To the best of our knowledge, this is the first study describing an ILC program for surface wipe sampling. ILC programs are already established in the domain of determination of HMPs in contaminated surface water and wastewater [23] as well as in closely related fields, such as therapeutic drug monitoring [15], immunology [16], microbiology [24], and the quality control (QC) of pharmaceuticals in drug development [25]. The parameters defined in this ILC program are challenging to directly extrapolate to those applied in the analysis of other HMPs in surface wipe samples. However, a recent review by Portilha‐Cunha et al. provided a comprehensive summary of performance parameters reported across a wide range of studies focusing on the determination of HMPs from surface wipe samples. While not all studies reported all three key parameters—accuracy, recovery, and precision—those that did demonstrated considerable variability. Reported accuracy values ranged from 61% to 133%, recovery rates varied widely from 25% to 120%, and precision rates were reported up to 16.2% [26]. Furthermore, the Occupational Safety and Health Administration guidelines recommend a recovery rate of at least 75%, with a preferred threshold of greater than 90% [27]. Given that this ILC program represents the first of its kind, the acceptance limits for all three parameters were established based on a rationale that considered both the feasibility of previously reported results and relevant literature benchmarks. Although no active ILC program currently exists for the analysis of HMPs in surface wipe samples, participation in an ILC is mandatory for ISO accreditation. Alternatively, another appropriate method must be used to ensure the validity of results (e.g., intralaboratory comparisons or comparison with reference materials) [13, 14]. Thus, establishing a feasible ILC program for laboratories on a global scale—considering the limited number of laboratories with validated methods for HMP analysis in surface wipe sampling—could enhance the performance of analytical methods and support compliance with ISO/IEC 17025:2017 and ISO 15189:2022 standards.

In addition, an ILC program designed to analyze HMPs on surfaces, which provides transparent correspondence about laboratories regarding their performance per HMP, allows healthcare organizations to identify laboratories equipped with internally validated analytical methods and compatible surface wipe sampling kits that meet established quality standards for methodological performance. Nevertheless, it is critical to recognize that performance may vary from laboratory to laboratory, even when certain limits are met. Therefore, healthcare organizations must recognize that longitudinal measurements may not be optimal if subsequent measurements are analyzed by different laboratories over time. Furthermore, it is recommended that surface wipe sampling be conducted by a limited number of qualified individuals, as the sampling process itself may contribute to increased uncertainty in the results.

As previously noted, this ILC program has the capacity to identify limitations in analytical methods and highlight potential underlying causes. However, an ILC program cannot identify the exact causes of parameters exceeding predetermined limits. Variations in measured concentrations, whether characterized by accuracy below or exceeding the predetermined limits, may arise from multiple factors, such as an internal standard that is either inadequately suited or too similar to the analyte, a limit of quantification determined during method validation that is higher than the lowest concentration included in the ILC program, the quality or composition of the QC, potential drift or degradation of analytes over time, the effect of the matrix, preparation of calibration standards, and environmental conditions during analysis [28, 29, 30, 31]. In the case of the low accuracy of paclitaxel quantification observed in Laboratory D, this is likely attributable to hydrolysis resulting from a lag time of several months between the preparation of the liquid samples and their subsequent analysis, due to a temporary malfunction of the liquid chromatograph‐tandem mass spectrometer. The liquid samples were diluted in an acetonitrile–water mixture (1:1, v/v). Paclitaxel is reported to degrade through hydrolysis in water under acidic, basic, and neutral conditions [32]. In contrast, the surface wipe samples were directly processed upon receipt using 100% acetonitrile as the final solvent. Because hydrolysis does not occur in 100% acetonitrile, the stability of paclitaxel in these samples was preserved. All samples were stored under identical conditions (−20°C for several months); however, differences in solvent composition likely influenced the stability of paclitaxel. This variation may explain the lower accuracy of paclitaxel quantification, particularly at lower concentrations, in the results obtained by Laboratory D. Notably, the concentrations that exceeded acceptable limits during this ILC program were predominantly at the lower end (Solution W).

Although recovery rates were achieved in 86% of cases overall, the relatively low recovery rates for etoposide, and particularly for paclitaxel, warrant attention to improve performance. While the exact cause remains unclear, the low recovery rates in surface wipe samples are likely attributable to insufficient absorption of HMPs during the wiping procedure or incomplete extraction from the wipe during the extraction process. Paclitaxel, in particular, demonstrated a low recovery rate in this study. This observation contrasts with previous studies, which reported recovery rates ranging from 78% to 98% [26]. However, it is important to consider that those earlier studies utilized highly organic extraction solutions, such as isopropyl alcohol or methanol, whereas the majority of laboratories participating in the present study employed extraction solutions with lower organic content, such as water or acidified water. It has been established that paclitaxel recovery is lower when a less organic or more aqueous solvent is used [33]. Laboratories could improve recoveries by exploring the use of different sampling materials and/or solvents used during the wiping procedure [26]. However, in this study, methods were applied that could detect multiple HMPs from one single wipe. In contrast, when multiple wipes from the same surface are necessary—due to the use of different analytical methods—there is a risk that certain HMPs may not be detected or may be detected at lower levels, as they may have already been removed by a previous wipe that was not designed to detect those specific HMPs. Additionally, the use of multiple separate methods for surface wipe sampling increases the complexity of the process, making it less efficient and more costly. A potential drawback of using a multianalyte method is the risk of reduced analytical performance for one or more HMPs. Therefore, each method must be carefully evaluated to ensure it achieves an adequate level of performance. Another factor is the variability in wiping by individuals, because it is known that the level of training and experience could influence the recovery [33]. Therefore, in our study, we chose to have the wiping performed by a single experienced laboratory technician.

Precision, as a secondary parameter, was calculated for Laboratories B, C, and D. Overall, the precision values were below the 20% in most cases for these three laboratories. The lower observed precision for paclitaxel, determined by Laboratory D, can be attributed to the same cause as the low accuracy (i.e., hydrolysis of paclitaxel in the liquid samples).

### 4.1 Strengths and Limitations

A notable strength of this ILC program was the utilization of Ph. Eur. CRS. These reference standards adhere to stringent standards concerning both qualitative and quantitative drug composition and are specifically designed for peak identification and system suitability testing in chromatography [34]. Furthermore, the spiking of the sample surfaces using an electronic microliter pipette resulted in highly accurate amounts of spiked HMPs. Additionally, the preparation of stock solutions and liquid samples was conducted by an independent laboratory technician who was not involved in other aspects of the program. Similarly, the spiking and wiping procedures were performed by a laboratory technician who remained unaware of the concentrations of the liquid samples. Lastly, all participating laboratories possessed knowledge only of the ILC program methodology, including the employed HMPs, but remained blinded regarding the specific concentrations of the HMPs utilized in the study.

This study, however, faced some limitations. First, the stability of the four concentrations was not assessed in detail. Following the preparation of the stock solution and the diluted solutions, these samples were stored in a refrigerator at temperatures between 2°C and 8°C for a maximum of 7 days. After spiking, the liquid samples, blank samples, and spiked samples were maintained at −20°C until being transported to the laboratories, where they were stored according to the standard operating procedures. It is well documented that the investigated HMPs exhibit stability for at least 1 week when stored under refrigeration or at −20°C. For instance, cyclophosphamide remains stable for up to 2 months at both 5°C and −20°C [35]. Kåredal et al. reported that cyclophosphamide, ifosfamide, etoposide, gemcitabine, and methotrexate maintain stability for 2 months at −20°C, while cyclophosphamide, ifosfamide, gemcitabine, and methotrexate remain stable for at least 10 days at 4°C. Etoposide exhibited a recovery rate of approximately 80% after 10 days at 4°C. Thus, the recovery rate remaining below 90% may be attributed to low extraction efficiency from the wipe following the extraction procedure, rather than to the chemical instability of etoposide [36]. Additionally, gemcitabine, cyclophosphamide, and paclitaxel in a acetonitrile–water mixture (1:1, v/v) within a concentration range of 5–100 ng/mL have been shown to be stable when stored in a refrigerator (2°C–8°C) [37]. Consequently, we have no reason to believe that the stability of the HMPs was compromised from the preparation of the stock and diluted solutions to the spiking phase or from storage after spiking until their delivery to the laboratories.

Secondly, precision was initially included as a parameter in the study design by calculating the RSD from triplicate analyses of the same sample. However, due to unforeseen circumstances, not all laboratories performed three replicate measurements for each sample. Incorporating precision into a future ILC program for the detection of HMPs on workplace surfaces could enhance the program's value by providing insights into the repeatability of precision, which refers to the variability in results obtained under identical conditions on the same sample. Moreover, this study represents only a single round of an ILC program. Beyond intrarun precision (i.e., within‐run precision), analyzing the same analyte under identical conditions, it would also be beneficial to include interrun precision (i.e., between‐run), analyzing the same analyte across different days or operators, in future ILC programs. Such an approach would offer a more comprehensive evaluation of method reproducibility [38].

Third, stainless steel was used as the surface for contamination and surface wipe sampling. Previous studies have shown that recovery rates vary depending on the surface type. However, the majority of studies on the determination of HMPs from surface wipe samples have utilized stainless steel as the sampling surface. Notably, stainless steel has demonstrated high recovery rates (up to 98%) whereas other surfaces, such as vinyl, exhibit substantially lower recovery rates (approximately 50%–60%) [26]. Given the variability in recovery across different surface types, we recommend the consistent use of a single surface material, preferably stainless steel, when implementing an ILC program for the determination of HMPs from surface wipe samples. This consistency will facilitate comparability of results across laboratories and measurement time points.

## 5 Conclusions

The ILC program examined in this study demonstrates suitability for independent, external evaluation of the performance of analytical methods for detecting HMPs in surface wipe samples, as it has the potential to reveal shortcomings in these methods. By implementing an ILC program for detecting HMPs from surface wipe samples, laboratories can determine the performance and potentially enhance their analytical methods. Further, healthcare organizations are enabled to identify laboratories that meet the expected quality standards for conducting analyses of HMPs in surface wipe samples. A future perspective is to implement a global ILC program by assigning a coordinating body to manage it effectively.

## Conflicts of Interest

The authors declare no conflicts of interest.

## Supporting information


**Figure S1.** Precision per HMP (cyclophosphamide, etoposide, gemcitabine, ifosfamide, methotrexate, and paclitaxel) and per laboratory (B, C, and D) for the four concentrations evaluated: 20 (Solution W, in green), 200 (Solution X, in blue), 2000 (Solution Y, in orange), and 5000 ng/mL (Solution Z, in yellow). The dashed horizontal lines represent the limit of 20%. The asterisk indicates that the concentrations obtained from the triplicate analyses were below the limit of quantification, making it impossible to calculate the RSD.
**Figure S2.** Precision per HMP (cyclophosphamide, etoposide, gemcitabine, ifosfamide, methotrexate, and paclitaxel) and per laboratory (B, C, and D) for the four concentrations, extracted from the wipe, evaluated: 0.05 (SWS‐W, in green), 0.5 (SWS‐X, in blue), 5 (Solution Y, in orange), and 12.5 ng/cm^2^ (SWS‐Z, in yellow). The dashed horizontal lines represent the limit of 20%.

**Table S1.** Overview of the validated SWS method per laboratory.
**Table S2.** Overview of the chemical reference substances utilized, along with their corresponding CAS numbers.
**Table S3.** Recovery (determined amount [ng/400 cm^2^]) per HMP (cyclophosphamide, etoposide, gemcitabine, ifosfamide, methotrexate, and paclitaxel) and per laboratory (A, B, C, and D) for SWS blanks (SWS‐BW, SWS‐X, SWS‐Y, and SWS‐Z).
**Table S4.** Accuracy (determined concentration [ng/mL] as percentage of the reference concentration) per HMP (cyclophosphamide, etoposide, gemcitabine, ifosfamide, methotrexate, and paclitaxel) and per laboratory (A, B, C, and D) for Solutions W, X, Y, and Z. Yellow indicates an accuracy below the limit (70%) and red above the limit (> 130%).
**Table S5.** Recovery (determined amount [ng/400 cm^2^] as percentage of the reference amount) per HMP (cyclophosphamide, etoposide, gemcitabine, ifosfamide, methotrexate, and paclitaxel) and per laboratory (A, B, C, and D) for SWS‐W, SWS‐X, SWS‐Y, and SWS‐Z. Yellow indicates a recovery below the limit (< 50%).
**Table S6.** Precision, expressed as the RSD derived from triplicate analyses of the same sample, per HMP (cyclophosphamide, etoposide, gemcitabine, ifosfamide, methotrexate, and paclitaxel) and per laboratory (C and D) for Solutions W, X, Y, and Z and SWS‐W, SWS‐X, SWS‐Y, and SWS‐Z. Red indicates a precision above the limit (20%). The precision of Laboratories A and B is not included due to the use of singlicate analyses (Laboratory A) and duplicate analyses (Laboratory B), which precluded the calculation of an RSD based on triplicate measurements of the same sample.

## Data Availability

Data are available upon reasonable request and based on the data use agreement policies of Amsterdam UMC hospital. Data from this study are available on request by sending an email message to the corresponding author.
