# Supplementary material for: Surface Wipe Sampling of Hazardous Medicinal Products: A European Interlaboratory Comparison Study
Source: Drug Test Anal. 2025 May 5;17(10):1955–64. doi: 10.1002/dta.3902 (PMC12489289; doi:10.1002/dta.3902)
Supplement: Supplementary file 1 — Figure S1. Precision per HMP (cyclophosphamide, etoposide, gemcitabine, ifosfamide, methotrexate, and paclitaxel) and per laboratory (B, C, and D) for the four concentrations evaluated: 20 (Solution W, in green), 200 (Solution X, in blue), 2000 (Solution Y, in orange), and 5000 ng/mL (Solution Z, in yellow). The dashed horizontal lines represent the limit of 20%. The asterisk indicates that the concentrations obtained from the triplicate analyses were below the limit of quantification, making it impossible to calculate the RSD. Figure S2. Precision per HMP (cyclophosphamide, etoposide, gemcitabine, ifosfamide, methotrexate, and paclitaxel) and per laboratory (B, C, and D) for the four concentrations, extracted from the wipe, evaluated: 0.05 (SWS‐W, in green), 0.5 (SWS‐X, in blue), 5 (Solution Y, in orange), and 12.5 ng/cm2 (SWS‐Z, in yellow). The dashed horizontal lines represent the limit of 20%. [file DTA-17-1955-s001.pdf]

SUPPLEMENTARY MATERIAL

Figures

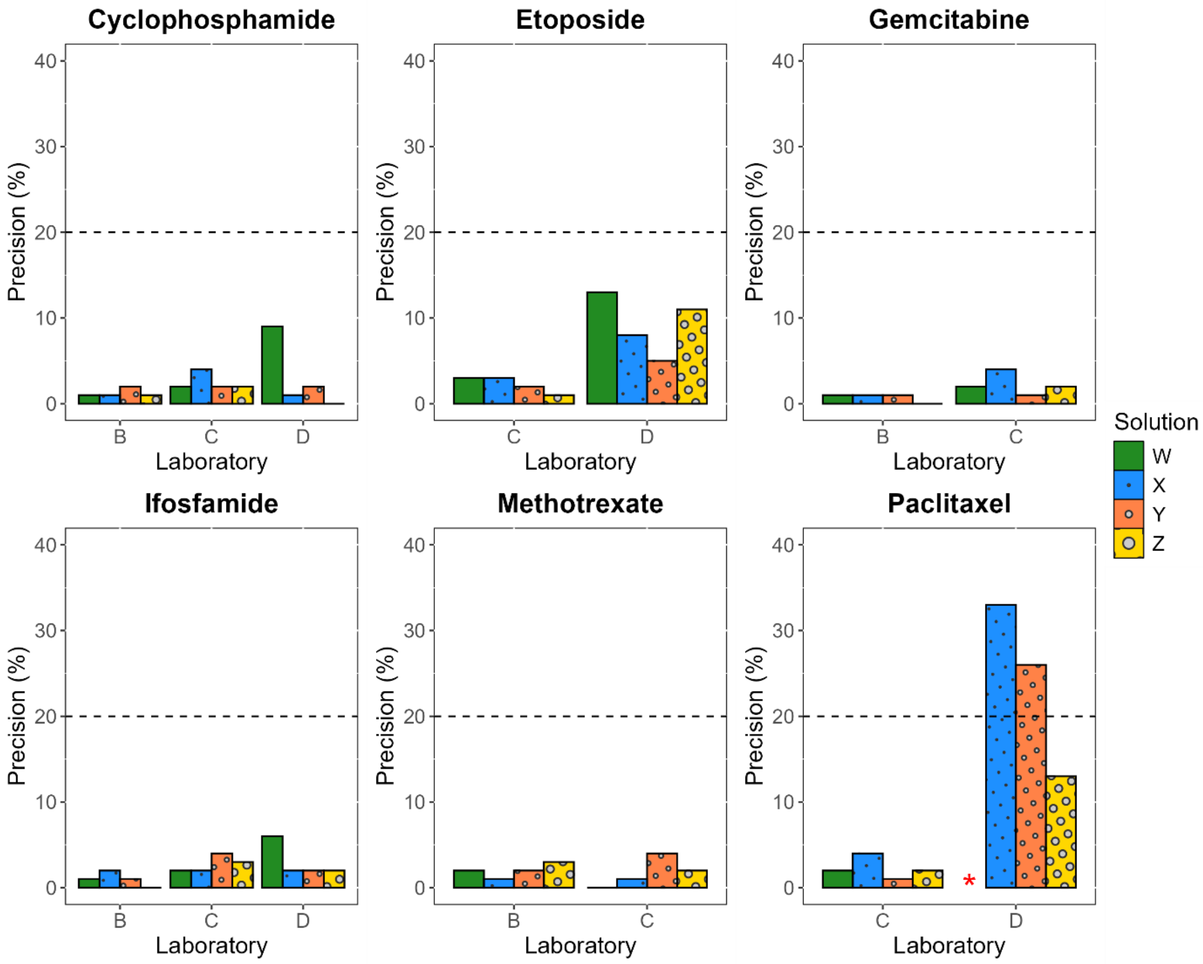

FIGURE S1 Precision per HMP (cyclophosphamide, etoposide, gemcitabine, ifosfamide, methotrexate and paclitaxel) and per laboratory (B, C and D) for the four concentrations evaluated: 20 ng/mL (solution W, in green), 200 ng/mL (solution X, in blue), 2000 ng/mL (solution Y, in orange) and 5000 ng/mL (solution Z, in yellow). The dashed horizontal lines represent the limit of 20%. The asterisk indicates that the concentrations obtained from the triplicate analyses were below the limit of quantification, making it impossible to calculate the RSD.

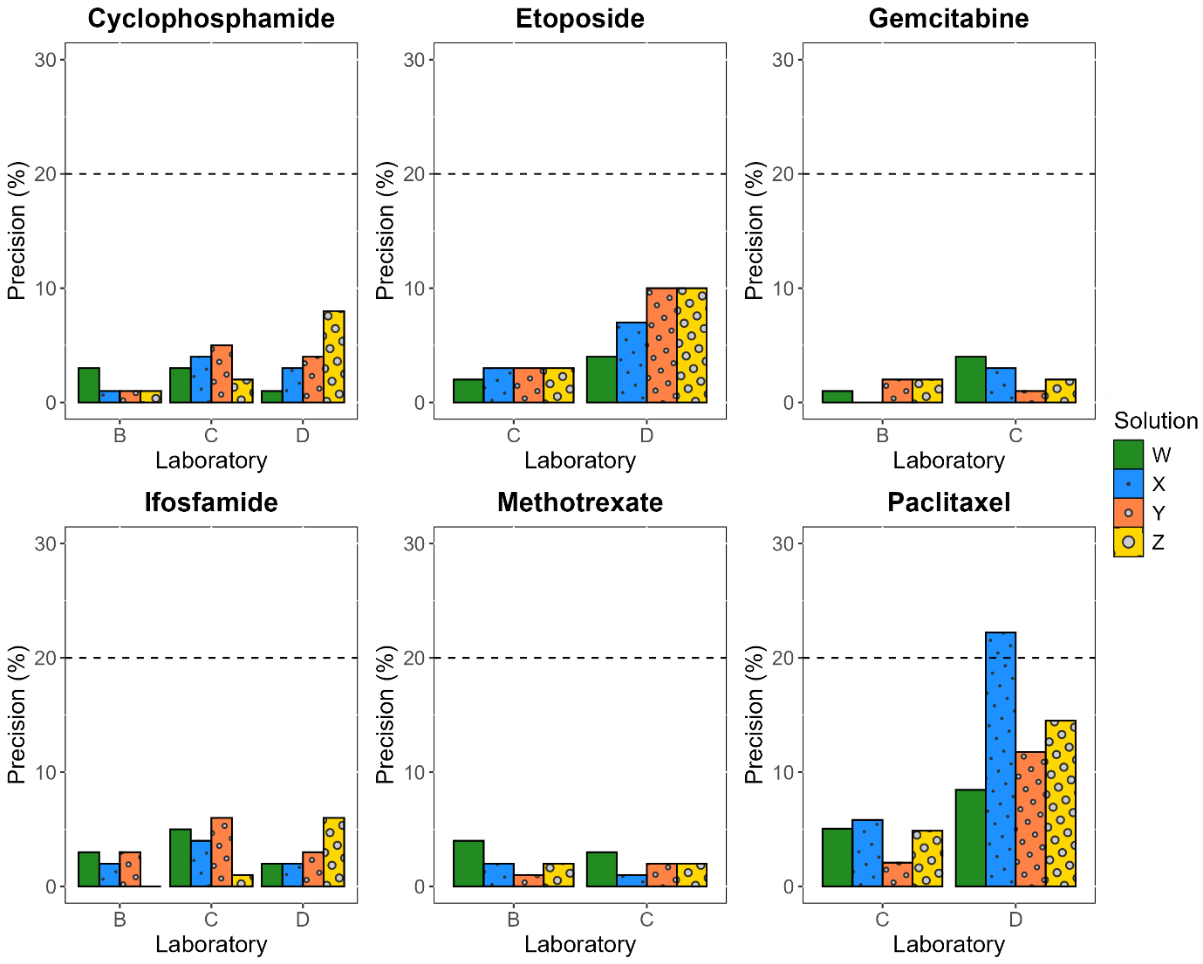

FIGURE S2 Precision per HMP (cyclophosphamide, etoposide, gemcitabine, ifosfamide, methotrexate and paclitaxel) and per laboratory (B, C and D) for the four concentrations, extracted from the wipe, evaluated: 0.05 ng/cm^2 (SWS-W, in green), 0.5 ng/cm^2 (SWS-X, in blue), 5 ng/cm^2 (solution Y, in orange) and 12.5 ng/cm^2 (SWS-Z, in yellow). The dashed horizontal lines represent the limit of 20%
