# Supplementary material for: Surface Wipe Sampling of Hazardous Medicinal Products: A European Interlaboratory Comparison Study
Source: Drug Test Anal. 2025 May 5;17(10):1955–64. doi: 10.1002/dta.3902 (PMC12489289; doi:10.1002/dta.3902)
Supplement: Supplementary file 2 — Table S1. Overview of the validated SWS method per laboratory. Table S2. Overview of the chemical reference substances utilized, along with their corresponding CAS numbers. Table S3. Recovery (determined amount [ng/400 cm2]) per HMP (cyclophosphamide, etoposide, gemcitabine, ifosfamide, methotrexate, and paclitaxel) and per laboratory (A, B, C, and D) for SWS blanks (SWS‐BW, SWS‐X, SWS‐Y, and SWS‐Z). Table S4. Accuracy (determined concentration [ng/mL] as percentage of the reference concentration) per HMP (cyclophosphamide, etoposide, gemcitabine, ifosfamide, methotrexate, and paclitaxel) and per laboratory (A, B, C, and D) for Solutions W, X, Y, and Z. Yellow indicates an accuracy below the limit (70%) and red above the limit (> 130%). Table S5. Recovery (determined amount [ng/400 cm2] as percentage of the reference amount) per HMP (cyclophosphamide, etoposide, gemcitabine, ifosfamide, methotrexate, and paclitaxel) and per laboratory (A, B, C, and D) for SWS‐W, SWS‐X, SWS‐Y, and SWS‐Z. Yellow indicates a recovery below the limit (< 50%). Table S6. Precision, expressed as the RSD derived from triplicate analyses of the same sample, per HMP (cyclophosphamide, etoposide, gemcitabine, ifosfamide, methotrexate, and paclitaxel) and per laboratory (C and D) for Solutions W, X, Y, and Z and SWS‐W, SWS‐X, SWS‐Y, and SWS‐Z. Red indicates a precision above the limit (20%). The precision of Laboratories A and B is not included due to the use of singlicate analyses (Laboratory A) and duplicate analyses (Laboratory B), which precluded the calculation of an RSD based on triplicate measurements of the same sample. [file DTA-17-1955-s002.docx]

**SUPPLEMENTARY MATERIAL**

**Tables**

**TABLE S1.** Overview of the validated SWS method per laboratory.

| Components of the SWS procedure | Laboratory A | Laboratory B | Laboratory C | Laboratory D |
| --- | --- | --- | --- | --- |
| Wipe material | The two layer wipes (20.5 x 21 cm) made of 100% pure cellulose fibers (7557, Kimberly-Clark, Koblenz, Germany). | Cotton swab (filled with 2 ml of water). | Non-woven swab (7.5 by 7.5 cm, four layers, viscose (70%) and polyester (30%), 30 g/m^2^). | Gauze parts (70% viscose and 30% polyester, 30 g/m^2^). |
| Wipe solution | 1 ml of diluted hydrochloric acid (pH 3) per tissue (3 mL per area). | 2 mL of water per 400 cm^2^. | 1 mL of 20 mM acetate buffer (pH 4) per area. | 2 mL of isopropanol per area. |
| Wipe procedure | Surfaces were thoroughly swept clean in vertical and horizontal strokes, changing the direction with every new tissue. The three tissues were combined to one sample, stored and transported in a 120 mL urine beaker. | Three times horizontally and vertically (alternating), applying pressure to the cotton swab to ensure the solution spreads evenly across the surface. | 1. Wipe the surface horizontally in a back-and-forth motion, covering the entire area. 2. Repeat the wipe vertically, perpendicular to the initial direction, to ensure full coverage. 3. Perform a final vertical wipe from the opposite side, ensuring thorough coverage from both vertical perspectives. | 1. Wipe one time horizontally: From left to right and then from right to left until reaching the bottom. 2. Wipe one time vertically: From top to bottom and then from bottom to top until reaching the left side of the area. 3. Wipe one time diagonally: Starting from the upper-left corner and ending at the lower-right corner.   Wipe using tweezers. |
| Extraction procedure | Addition of 30 mL of deionised water (adjusted to pH 3 with HCl) and shaken manually very intensively for approx. 15 seconds. | Extraction of the solvent by centrifugation the wipe. | Extraction of swabs: 25 mL of acetate buffer (20 mM) in a cooled ultrasonic bath for 45 min. | Addition of 1 mL acetonitrile (extraction solvent), 20 min agitation in an ultrasonic bath, evaporation to dryness, reconstitution in 200 µL acetonitrile. |
| Analytical procedure | LC-MS/MS | LC-MS/MS | LC-MS/MS | LC-MS/MS |

**TABLE S2.** Overview of the chemical reference substances utilized, along with their corresponding CAS numbers.

| Chemical reference substance | CAS number |
| --- | --- |
| Cyclophosphamide CRS | 6055-19-2 |
| Etoposide CRS | 33419-42-0 |
| Gemcitabine hydrochloride CRS | 122111-03-9 |
| Ifosfamide CRS | 3778-73-2 |
| Methotrexate CRS | 59-05-2 |
| Paclitaxel CRS | 33069-62-4 |

**TABLE S3.** Recovery (determined amount (ng/400 cm^2^) per HMP (cyclophosphamide, etoposide, gemcitabine, ifosfamide, methotrexate and paclitaxel) and per laboratory (A, B, C and D) for SWS blanks (SWS-BW, SWS-X, SWS-Y and SWS-Z).

|  |  | SWS-BW | SWS-BX | SWS-BY | SWS-BZ |
| --- | --- | --- | --- | --- | --- |
|  |  | Amount (ng/400 cm^2^) | Amount (ng/400 cm^2^) | Amount (ng/400 cm^2^) | Amount (ng/400 cm^2^) |
| Cyclophosphamide | A | <0.0075 | <0.0075 | <0.0075 | <0.0075 |
|  | B | <0.0125 | <0.0125 | <0.0125 | <0.0125 |
|  | C | <0.001 | <0.001 | <0.001 | <0.001 |
|  | D | n.d. | n.d. | n.d. | n.d. |
| Etoposide | A | <0.0075 | <0.0075 | <0.0075 | <0.0075 |
|  | B | n.a. | n.a. | n.a. | n.a. |
|  | C | <0.004 | <0.004 | <0.004 | <0.004 |
|  | D | n.d. | n.d. | n.d. | n.d. |
| Gemcitabine | A | <0.0075 | <0.0075 | <0.0075 | <0.0075 |
|  | B | <0.0125 | <0.0125 | <0.0125 | <0.0125 |
|  | C | <0.002 | <0.002 | <0.002 | <0.002 |
|  | D | n.a. | n.a. | n.a. | n.a. |
| Ifosfamide | A | <0.0075 | <0.0075 | <0.0075 | <0.0075 |
|  | B | <0.0125 | <0.0125 | <0.0125 | <0.0125 |
|  | C | <0.002 | <0.002 | <0.002 | <0.002 |
|  | D | n.d. | n.d. | n.d. | n.d. |
| Methotrexate | A | <0.0075 | <0.0075 | <0.0075 | <0.0075 |
|  | B | <0.0125 | <0.0125 | <0.0125 | <0.0125 |
|  | C | <0.002 | <0.002 | <0.002 | <0.002 |
|  | D | n.a. | n.a. | n.a. | n.a. |
| Paclitaxel | A | <0.02 | <0.02 | <0.02 | <0.02 |
|  | B | n.a. | n.a. | n.a. | n.a. |
|  | C | <0.004 | <0.004 | <0.004 | <0.004 |
|  | D | n.d. | n.d. | n.d. | n.d. |

Abbreviations: n.a., not applicable; n.d., not detectable

**TABLE S4.** Accuracy (determined concentration (ng/mL) as percentage of the reference concentration) per HMP (cyclophosphamide, etoposide, gemcitabine, ifosfamide, methotrexate and paclitaxel) and per laboratory (A, B, C and D) for solution W, X, Y and Z. Yellow indicates an accuracy below the limit (70%) and red above the limit (>130%).

|  |  | W (20 ng/mL) | | X (200 ng/mL) | | Y (2000 ng/mL) | | Z (5000 ng/mL) | |
| --- | --- | --- | --- | --- | --- | --- | --- | --- | --- |
|  |  | Concentration (ng/ml) | Accuracy (%) | Concentration (ng/ml) | Accuracy (%) | Concentration (ng/ml) | Accuracy (%) | Concentration (ng/ml) | Accuracy (%) |
| Cyclophosphamide | A | 28 | 140% | 222 | 111% | 2235 | 112% | 5812 | 116% |
|  | B | 17 | 83% | 164 | 82% | 1690 | 85% | 4160 | 83% |
|  | C | 19 | 95% | 218 | 109% | 1841 | 92% | 4368 | 87% |
|  | D | 11 | 55% | 184 | 92% | 1773 | 89% | 4422 | 88% |
| Etoposide | A | 27 | 135% | 220 | 110% | 1842 | 92% | 4164 | 83% |
|  | B | n.a. | n.a. | n.a. | n.a. | n.a. | n.a. | n.a. | n.a. |
|  | C | 16 | 78% | 226 | 113% | 1657 | 83% | 4028 | 81% |
|  | D | 23 | 115% | 156 | 78% | 1707 | 85% | 4339 | 87% |
| Gemcitabine | A | 26 | 130% | 190 | 95% | 1986 | 99% | 5308 | 106% |
|  | B | 15 | 75% | 151 | 76% | 1434 | 72% | 3448 | 69% |
|  | C | 17 | 86% | 197 | 98% | 1657 | 83% | 4028 | 81% |
|  | D | n.a. | n.a. | n.a. | n.a. | n.a. | n.a. | n.a. | n.a. |
| Ifosfamide | A | 23 | 115% | 202 | 101% | 1896 | 95% | 4858 | 97% |
|  | B | 17 | 85% | 177 | 89% | 1794 | 90% | 4517 | 90% |
|  | C | 19 | 94% | 210 | 105% | 1783 | 89% | 3710 | 74% |
|  | D | 16 | 80% | 177 | 89% | 1710 | 86% | 4493 | 90% |
| Methotrexate | A | 30 | 150% | 234 | 117% | 2289 | 114% | 5578 | 112% |
|  | B | 23 | 113% | 226 | 113% | 2192 | 110% | 5286 | 106% |
|  | C | 20 | 101% | 226 | 113% | 1926 | 96% | 4301 | 86% |
|  | D | n.a. | n.a. | n.a. | n.a. | n.a. | n.a. | n.a. | n.a. |
| Paclitaxel | A | 29 | 145% | 273 | 137% | 2734 | 137% | 4858 | 97% |
|  | B | n.a. | n.a. | n.a. | n.a. | n.a. | n.a. | n.a. | n.a. |
|  | C | 17.3 | 86% | 197 | 98% | 1737 | 87% | 3710 | 74% |
|  | D | 0 | 0% | 76 | 38% | 1256 | 63% | 3972 | 79% |

Abbreviations: n.a., not applicable

**TABLE S5.** Recovery (determined amount (ng/400 cm^2^) as percentage of the reference amount) per HMP (cyclophosphamide, etoposide, gemcitabine, ifosfamide, methotrexate and paclitaxel) and per laboratory (A, B, C and D) for SWS-W, SWS-X, SWS-Y and SWS-Z. Yellow indicates a recovery below the limit (<50%).

|  |  | SWS-W (0.05 ng/400 cm^2^) | | SWS-X (0.5 ng/400 cm^2^) | | SWS-Y (5 ng/400 cm^2^) | | SWS-Z (12.5 ng/400 cm^2^) | |
| --- | --- | --- | --- | --- | --- | --- | --- | --- | --- |
|  |  | Amount (ng/400 cm^2^) | Recovery | Amount (ng/400 cm^2^) | Recovery | Amount (ng/400 cm^2^) | Recovery | Amount (ng/400 cm^2^) | Recovery |
| Cyclophosphamide | A | 0.065 | 130% | 0.58 | 115% | 5.3 | 105% | 15.8 | 126% |
|  | B | 0.045 | 90% | 0.44 | 89% | 4.1 | 81% | 10.0 | 80% |
|  | C | 0.042 | 85% | 0.41 | 82% | 4.4 | 88% | 10.5 | 84% |
|  | D | 0.028 | 56% | 0.25 | 50% | 3.4 | 69% | 8.6 | 69% |
| Etoposide | A | 0.040 | 80% | 0.38 | 75% | 4.8 | 95% | 10.8 | 86% |
|  | B | n.a. | n.a. | n.a. | n.a. | n.a. | n.a. | n.a. | n.a. |
|  | C | 0.045 | 90% | 0.38 | 76% | 2.9 | 59% | 6.9 | 55% |
|  | D | 0.020 | 40% | 0.18 | 36% | 3.1 | 62% | 8.1 | 65% |
| Gemcitabine | A | 0.048 | 95% | 0.53 | 105% | 4.5 | 90% | 13.0 | 104% |
|  | B | 0.038 | 77% | 0.38 | 77% | 3.5 | 69% | 8.6 | 69% |
|  | C | 0.045 | 91% | 0.40 | 80% | 4.1 | 83% | 10.2 | 82% |
|  | D | n.a. | n.a. | n.a. | n.a. | n.a. | n.a. | n.a. | n.a. |
| Ifosfamide | A | 0.048 | 95% | 0.50 | 100% | 4.8 | 95% | 13.8 | 110% |
|  | B | 0.041 | 82% | 0.46 | 92% | 4.5 | 90% | 11.1 | 89% |
|  | C | 0.043 | 85% | 0.39 | 79% | 4.2 | 84% | 10.4 | 83% |
|  | D | 0.032 | 63% | 0.27 | 54% | 3.7 | 73% | 9.2 | 73% |
| Methotrexate | A | 0.050 | 100% | 0.43 | 85% | 4.8 | 95% | 13.0 | 104% |
|  | B | 0.052 | 104% | 0.60 | 119% | 5.4 | 107% | 12.5 | 100% |
|  | C | 0.042 | 84% | 0.39 | 78% | 4.3 | 86% | 9.8 | 78% |
|  | D | n.a. | n.a. | n.a. | n.a. | n.a. | n.a. | n.a. | n.a. |
| Paclitaxel | A | 0.000 | 0% | 0.00 | 0% | 0.0 | 0% | 0.0 | 0% |
|  | B | n.a. | n.a. | n.a. | n.a. | n.a. | n.a. | n.a. | n.a. |
|  | C | 0.025 | 50% | 0.27 | 55% | 2.6 | 53% | 4.7 | 38% |
|  | D | 0.018 | 36% | 0.25 | 50% | 4.3 | 86% | 11.9 | 96% |

Abbreviations: n.a., not applicable

**TABLE S6.** Precision, expressed as the RSD derived from triplicate analyses of the same sample, per HMP (cyclophosphamide, etoposide, gemcitabine, ifosfamide, methotrexate and paclitaxel) and per laboratory (C and D) for solution W, X, Y and Z and SWS-W, SWS-X, SWS-Y and SWS-Z. Red indicates a precision above the limit (20%). The precision of laboratories A and B is not included due to the use of singlicate analyses (laboratory A) and duplicate analyses (laboratory B), which precluded the calculation of an RSD based on triplicate measurements of the same sample.

|  |  | Precision (%) | | | | | | | |
| --- | --- | --- | --- | --- | --- | --- | --- | --- | --- |
|  |  | W | X | Y | Z | SWS-W | SWS-X | SWS-Y | SWS-Z |
| Cyclophosphamide | B | 1% | 1% | 2% | 1% | 3% | 1% | 1% | 1% |
|  | C | 2% | 4% | 2% | 2% | 3% | 4% | 5% | 2% |
|  | D | 9% | 1% | 2% | 0% | 1% | 3% | 4% | 8% |
| Etoposide | B | n.a. | n.a. | n.a. | n.a. | n.a. | n.a. | n.a. | n.a. |
|  | C | 3% | 3% | 2% | 1% | 2% | 3% | 3% | 3% |
|  | D | 13% | 8% | 5% | 11% | 4% | 7% | 10% | 10% |
| Gemcitabine | B | 1% | 1% | 1% | 0% | 1% | 0% | 2% | 2% |
|  | C | 2% | 4% | 1% | 2% | 4% | 3% | 1% | 2% |
|  | D | n.a. | n.a. | n.a. | n.a. | n.a. | n.a. | n.a. | n.a. |
| Ifosfamide | B | 1% | 2% | 1% | 0% | 3% | 2% | 3% | 0% |
|  | C | 2% | 2% | 4% | 3% | 5% | 4% | 6% | 1% |
|  | D | 6% | 2% | 2% | 2% | 2% | 2% | 3% | 6% |
| MTX | B | 2% | 1% | 2% | 3% | 4% | 2% | 1% | 2% |
|  | C | 0% | 1% | 4% | 2% | 3% | 1% | 2% | 2% |
|  | D | n.a. | n.a. | n.a. | n.a. | n.a. | n.a. | n.a. | n.a. |
| Paclitaxel | B | n.a. | n.a. | n.a. | n.a. | n.a. | n.a. | n.a. | n.a. |
|  | C | 2% | 4% | 1% | 2% | 5% | 6% | 2% | 5% |
|  | D | n.a.* | 33% | 26% | 13% | 8% | 22% | 12% | 15% |

*Note:* *The concentrations obtained from the triplicate analyses were below the limit of quantification, making it impossible to calculate the RSD.

Abbreviations: n.a., not applicable
